# Differential Processing of *let-7*a Precursors Influences RRM2 Expression and Chemosensitivity in Pancreatic Cancer: Role of LIN-28 and SET Oncoprotein

**DOI:** 10.1371/journal.pone.0053436

**Published:** 2013-01-15

**Authors:** Yangzom Doma Bhutia, Sau Wai Hung, Madeline Krentz, Dimal Patel, Dylan Lovin, Radhika Manoharan, J. Michael Thomson, Rajgopal Govindarajan

**Affiliations:** 1 Department of Pharmaceutical and Biomedical Sciences, The University of Georgia, Athens, Georgia, United States of America; 2 Cancer Biology, Vanderbilt-Ingram Cancer Center, Vanderbilt University Medical School, Nashville, Tennessee, United States of America; Complutense University, Spain

## Abstract

Overexpression of ribonucleotide reductase subunit M2 (RRM2), involved in deoxyribonucleotide synthesis, drives the chemoresistance of pancreatic cancer to nucleoside analogs (e.g., gemcitabine). While silencing RRM2 by synthetic means has shown promise in reducing chemoresistance, targeting endogenous molecules, especially microRNAs (miRNAs), to advance chemotherapeutic outcomes has been poorly explored. Based on computational predictions, we hypothesized that the *let-7* tumor suppressor miRNAs will inhibit RRM2-mediated gemcitabine chemoresistance in pancreatic cancer. Reduced expression of the majority of *let-7* miRNAs with an inverse relationship to RRM2 expression was identified in innately gemcitabine-resistant pancreatic cancer cell lines. Direct binding of *let-7* miRNAs to the 3′ UTR of RRM2 transcripts identified post-transcriptional regulation of RRM2 influencing gemcitabine chemosensitivity. Intriguingly, overexpression of human precursor-*let-7* miRNAs led to differential RRM2 expression and chemosensitivity responses in a poorly differentiated pancreatic cancer cell line, MIA PaCa-2. Defective processing of *let-7a* precursors to mature forms, in part, explained the discrepancies observed with *let-7a* expressional outcomes. Consistently, the ratios of mature to precursor *let-7a* were progressively reduced in gemcitabine-sensitive L3.6pl and Capan-1 cell lines induced to acquire gemcitabine resistance. Besides known regulators of *let-7* biogenesis (e.g., LIN-28), short hairpin RNA library screening identified several novel RNA binding proteins, including the SET oncoprotein, to differentially impact *let-7* biogenesis and chemosensitivity in gemcitabine-sensitive versus -resistant pancreatic cancer cells. Further, LIN-28 and SET knockdown in the cells led to profound reductions in cellular proliferation and colony-formation capacities. Finally, defective processing of *let-7a* precursors with a positive correlation to RRM2 overexpression was identified in patient-derived pancreatic ductal adenocarcinoma (PDAC) tissues. These data demonstrate an intricate post-transcriptional regulation of RRM2 and chemosensitivity by *let-7a* and that the manipulation of regulatory proteins involved in *let-7a* transcription/processing may provide a mechanism for improving chemotherapeutic and/or tumor growth control responses in pancreatic cancer.

## Introduction

Ribonucleotide reductase (RR) is a rate-limiting enzyme for cell replication which catalyzes the reduction of ribonucleotides to deoxyribonucleotides during DNA synthesis. It is overexpressed in a number of solid tumors including pancreatic [1]. Accumulating evidence suggests that RR acts as a positive determinant for tumor cell proliferation and metastasis as well as the development of chemoresistance to nucleoside analogs used for treating pancreatic cancer (e.g., gemcitabine, capecitabine, 5-fluorouracil) [2]–[6]. RR activity is regulated during S-phase of the cell cycle primarily by transcriptional activation of one of its non-identical subunits, called RRM2 [7], [8]. RRM2 expression has been shown to be induced in chemoresistant cells by gene amplification, transcriptional activation, and perhaps other unidentified mechanisms [5], [9]. Recent studies have shown that exogenous manipulations of RRM2 expression by siRNA or antisense oligonucleotides improve chemosensitivity in pancreatic cancer [10], [11].

Although downmodulation of RRM2 by synthetic means (e.g., siRNA) has shown potential in decreasing tumor growth and gemcitabine chemoresistance, the possibilities of manipulating endogenous molecules to improve gemcitabine responses and perhaps improving therapeutic outcomes in pancreatic cancer have never been explored. MicroRNAs (miRNAs), endogenously-expressed ∼22-nt long RNAs capable of post-transcriptionally silencing target gene expressions, offer several advantages in this regard. For instance, the large number of miRNAs in the human genome and their diverse targets [12] allow selection of miRNA(s) not only to improve chemosensitization but to also favorably impact many gene regulatory networks involved in aspects such as tumor growth, invasion, cancer stem cell survival, etc. [13], [14]. Further, since miRNAs are frequently downregulated in cancers [15], [16], reestablishing their expression is likely to facilitate synergistic growth-control responses with chemotherapeutic agents. In addition, expanding the understanding of miRNA gene regulation will provide opportunities for manipulating their expression with small molecules without the complexity of synthetic oligonucleotide delivery into tumors.

In searching for putative miRNA inhibitors of RRM2 by computational miRNA target prediction algorithms, we found the *let-7* family of tumor suppressor miRNAs to possess a seed match for base pairing with the 3′ UTR of RRM2 (context score percentile: 94; TargetScanHuman 5.1). Consistently, earlier studies have implicated a causal relationship between *let-7* and RRM2, identifying downregulation of many *let-7* family members in RRM2-overexpressing, gemcitabine-resistant pancreatic cancer cells or a reduction in RRM2 expression after *let-7* overexpression [17], [18]. Further, overexpression of *let-7* was found to increase the radiosensitization of pancreatic tumor cells [19], while inhibition of RRM2 was identified to sensitize pancreatic tumors to ultraviolent radiation [20], [21]. Recently, forced expression of *let-7* miRNAs was shown to inhibit pancreatic cancer cell proliferation *in vitro* but not tumor growth *in vivo* suggesting the presence of complex functional ramifications [22]. Hence, to study the potential interplay between *let-7* and RRM2 and to further explore the opportunity of utilizing *let-7* for pancreatic cancer therapeutics, we sought to determine the direct impact of the human *let-7* family on RRM2-mediated inherent gemcitabine resistance. Here we report an intricate regulation of RRM2 expression and gemcitabine chemosensitization by *let*-7*a* precursors and identify that the miRNA transcriptional/processing machinery involved in mature *let-7a* biogenesis is likely to act as a crucial factor when considering *let*-7a-based therapeutics for pancreatic cancer.

## Materials and Methods

### Tumor RNA and Tissues

Total RNA from 10 PDAC tissues and 2 normal pancreatic tissues were procured from Asterand (Detroit, MI). The demographic and clinical information available with the RNA samples are shown in *Table S1*. Six PDAC tissue samples along with the matched normal adjacent tissues were procured from the National Disease Research Interchange (Philadelphia, PA) [23]. NDRI obtains written consents from the sources. The procurement and use of these human tissues for this research was done in accordance with the University of Georgia Institutional Review Board. The Board has determined that the use of human biological tissues in this research does not meet the criteria for research involving human subjects per 45 CFR 46.102, and therefore does not require human subject approval by the Board.

### Reagents

Gemcitabine was obtained from ChemieTek (Indianapolis, IN). Fetal bovine serum (FBS) was from PAA Laboratories, Inc. (Ontario, Canada). 4′,6′-diamidino-2-phenylindole (DAPI), dimethylsulfoxide (DMSO), 3-(4,5-dimethylthiazol-2-yl)-2,5-diphenyltetrazolium bromide (MTT), propidium iodide, ethylene glycol bis(2-aminoethyl ether) tetraacetic acid (EGTA), phenylmethanesulfonyl fluoride (PMSF), N-ethyl maleimide (NEM), sodium orthovanadate (Na_2_VO_4_), and iodoacetamide were obtained from Sigma Aldrich (St. Louis, MO). The bicinchoninic acid (BCA) protein assay reagent and West Pico Chemiluminiscent substrate were from Pierce Chemical (Rockford, IL). Fluorescent anti-fade mounting reagent and Vybrant DyeCycle green were obtained from Molecular Probes (Invitrogen, Carlsbad, CA). Plasticware for cell culture was obtained from Corning (Corning, NY). All cell culture media were purchased from Mediatech (Manassas, VA) except for the human keratinocyte basal medium which was procured from Molecular Probes.

### Antibodies

The anti-human goat polyclonal RRM1 (T-16), RRM2 (E-16), dCK (L-19), hCNT3 (C-15), and SET/I2PP2A (E-15) antibodies as well as the mouse monoclonal hnRNP-A1 (4B10) antibody were obtained from Santa Cruz Biotechnology (Santa Cruz, CA). The details of hENT1, hENT2, hCNT1, hCNT2, and β-actin antibodies were described earlier [23], [24]. The anti-human rabbit polyclonal CDA (ab56053) and anti-human mouse monoclonal KHSRP antibodies were obtained from Abcam (Cambridge, MA). The anti-human rabbit polyclonal LIN-28 antibody was obtained from Cell Signaling Technology (Danvers, MA).

### Constructs

RRM2 cDNA without the 3′ UTR region was constructed from RRM2 truclone (RRM2 (NM_001165931) Human cDNA Clone; Product ID: SC326997; Origene, MD) using PCR-based methods. The pmiRGLO-G-FUD construct was initially obtained from Promega (Madison, WI) and modified to contain GFP fused with the firefly luciferase gene, thus making it a dual reporter assay. The promoter was also removed and exchanged for the CMV promoter. As a reporter for processing, constructs that contained ∼50 base pairs upstream and downstream of the *let-7a-1* (pmirGLO-GFP/luc-pre-*let-7a-1*) and *let-7b* (pmiR-glo-GFP/luc-pre-*let-7b*) microRNAs were made and cloned downstream of the GFP-Luc 3′ UTR. When properly processed, the cleavage of the microRNA destabilizes the GFP/Luc mRNA leading to a reduction in both proteins.

### Cell Culture, Immunocytochemistry, MTT Cytotoxicity, Flow Cytometry, Colony Formation, and Real-time PCR Assays

These procedures were performed as described earlier [23], [24]. TaqMan primers and probes for RRM2 (Hs00357251_g1) were obtained from Applied Biosystems (Foster City, CA).

### miRNA Detection

Total RNA isolation and cDNA synthesis were performed using the *mir*Vana miRNA Isolation Kit and the TaqMan® MicroRNA Reverse Transcription Kit (Applied Biosystems), respectively, as per the manufacturers’ instructions. Relative quantification of RNA transcripts was performed as described earlier [23], [24]. Validated TaqMan primers and probes for *let-7a* (hsa000377), *let-7b* (hsa002619), *let-7c* (hsa000379), *let-7d* (hsa002283), *let-7e* (hsa002406), *let-7f* (hsa000382), *let-7g* (hsa002282), *let-7i* (hsa002221), pri-*let-7a-1* (Hs03302533_pri), pri-*let-7a-2* (Hs03302539_pri), and pri-*let-7a-3* (Hs03302546_pri) purchased from Applied Biosystems were used.

### Generation of Gemcitabine-resistant Pancreatic Tumor Cells

Cells were exposed to increasing concentrations (5–20 nM) of gemcitabine for 5–6 weeks per concentration.

### Western Blotting

Western blotting analysis was performed as described earlier [23], [24] except for the following modifications. Total cell lysates were prepared in lysis buffer containing 10 mM Tris, 1mM EGTA, 1 mM PMSF, 10 mM sodium fluoride, 10 mM NEM, 10 mM Na_2_VO_4_, 10 mM iodoacetamide, 0.5% Triton-X 100, and a protease inhibitor cocktail (Roche, Mannheim, Germany). The pH of the lysis buffer was maintained at 7.4. After assessing protein content with the BCA Protein Assay Kit, 50 µg/lane were loaded and electrophoretically separated on 10% polyacrylamide sodium dodecyl sulphate (SDS-PAGE) gels and transferred at 150 V for 1 h or 20 V overnight to a polyvinylidine fluoride (PVDF) membrane (Millipore, Billerica, MA). Antibodies against the metabolizing enzymes (RRM1, RRM2, dCK, CDA) and nucleoside transporters (hCNT1, hCNT3, ENT1, ENT2) [24] were used in 1∶500–1000 dilutions. Densitometric analyses were performed as described before [23].

### Generation of MIA PaCa-2 Stable Cells Overexpressing Pre-*let-7* Members

FIV-let-7 constructs (*let-7a-2*, *let-7a-3*, *let-7b*, *let-7c*, *let-7e*, *let-7f-1*, *let-7f-2*, *let-7g*, and*let-7i*) from GeneCopoeia (Rockville, MD) and HIV-let-7 constructs (let-7a-1 and let-7d) from System Biosciences (Mountain View, CA) were used. Lentiviral packaging cells (293Ta; GeneCopoeia) were seeded at a density of 1.3–1.5×10^6^ in 10 cm dishes containing 10 ml of DMEM supplemented with 10% heat-inactivated FBS. Cells were allowed to reach 70–80% confluency at the time of transfection of the above mentioned constructs. Viral infections were performed as per the manufacturer’s instructions. Briefly, 2.5 µg of the expression DNA clone and 5 µl (0.5 µg/µl) of Lenti-Pac FIV/HIV mix were diluted into 200 µl of Opti-MEM (Invitrogen). In a separate tube, 15 µl of EndoFectin Lenti was also diluted in 200 µl of Opti-MEM. The diluted EndoFectin Lenti reagent was then added drop-wise to the DNA solution and vortexed. The mixture was then incubated for 10–25 min at room temperature to allow the DNA-Endofectin complex to form. The DNA-Endofectin Lenti complex was directly added to each dish of 293Ta cells. The dishes were gently swirled to distribute the complex and then incubated overnight in a CO_2_ incubator at 37°C. The cells were replaced with fresh DMEM medium after 24 h, and lentiviruses were harvested and filtered at 24–48 h post-transfection. For the transduction of packaged lentiviral expression clones, MIA PaCa-2 cells were seeded in 10 cm dishes 24 h prior to viral infection. They were subsequently infected with 3 mL of the viral suspension in solution with 8 µg/mL of polybrene. The cells were then incubated overnight in a CO_2_ incubator at 37°C after which they were supplemented with fresh media. After a 24 h growth period, cultures infected with Zeocin-resistant genes (pMIF-eGFP-Zeo) were selected with 400 µg/ml Zeocin (Invitrogen), and those infected with puromycin-resistant genes (pEZX-MR04) were selected with 10 µg/ml puromycin.

### shRNA-based Screening for Putative RNA Processing Proteins

96 shRNA constructs from Open Biosystems (Thermo Fisher Scientific, Huntsville, AL) were obtained from the Genome Sciences Resource at Vanderbilt University. The lentiviral packaging cell line (Phoenix) was seeded at a density of 1×10^6^ in 6 cm dishes containing 5 ml of DMEM supplemented with 10% heat-inactivated FBS. Cells were grown to 70–80% confluency at the time of transfection with the shRNA constructs. Viral infections were performed as per the manufacturer’s instructions. Briefly, 1 µg of the expression DNA clone, 0.75 µg of the packaging vector (pCMV∼DR7.74psPAX2) and 0.3 µg of the envelope vector (pMD2.G) were mixed and added to a tube containing 200 µl of Opti-MEM (Invitrogen) and 6 µl of Fugene 6 (Roche). The mixture was then incubated for 25 min at room temperature after which it was directly added to each dish of Phoenix cells. The dishes were gently swirled to distribute the complex and then incubated overnight in a CO_2_ incubator at 37°C. The cells were replaced with fresh DMEM medium after 24 h, and lentiviruses were harvested and filtered at 24–48 h post-transfection. For the transduction of packaged lentiviral expression clones, MIA PaCa-2 and L3.6pl cells were seeded in 6-well clusters 24 h prior to viral infection. They were subsequently infected with 1 mL of the viral suspension in solution with 4 µg/mL of polybrene. The cells were then incubated overnight in a CO_2_ incubator at 37°C after which they were supplemented with fresh media. After a 24 h growth period, cells were selected with 0.5 and 0.1 µg/mL of puromycin for MIA PaCa-2 and L3.6pl, respectively. Several stable clones were screened by microscopy for GFP coexpression.

### Target *In Vitro* Reporter Assay

For luciferase binding assays, 293Ta cells were seeded on a 24-well cluster (5×10^3^ cells/well) and transduced with various let-7 precursors using the lentiviral gene transfer method (as described earlier). Twenty-four h after seeding, cells were replenished with fresh media and transfected with 1 µg of the control or RRM2 3′ UTR target vector (ID: HmiT053958; GeneCopoeia), 100 µl of Opti-MEM (Invitrogen) and 3 µl FuGeneHD transfection reagent (Roche) as per the manufacturer’s protocol. Thirty-six h after transfection, cells were lysed by the passive lysis procedure and handled as described in the Dual-Luciferase Assay Kit (Promega). Cell lysates were centrifuged at 10,000 rpm for 30 s, and the luciferase assay was performed with the clear supernatant transferred to a black 96-well microtiter plate (Greiner Bio–One, Monroe, NC). Twenty µl of cell lysate was mixed with 100 µl of LARII solution to begin the reaction, and 100 µl of Stop and Glo reagent was used to simultaneously quench luciferase and start the renilla reaction. Luciferase and renilla luminescence were quantified at 100 nm, and each experiment was repeated three times. Data were expressed as mean±SD.

### Statistical Analysis

The student's t test was used to identify significant differences, and each experiment was repeated at least three times. Unless otherwise indicated, *p*<0.05, *p*<0.01, and *p*<0.001 compared with control conditions were represented by one, two, and three asterisks, respectively.

## Results

### RRM2 and *let-7* are Inversely Expressed in Human Pancreatic Cancer Cells

**Figure 1:**
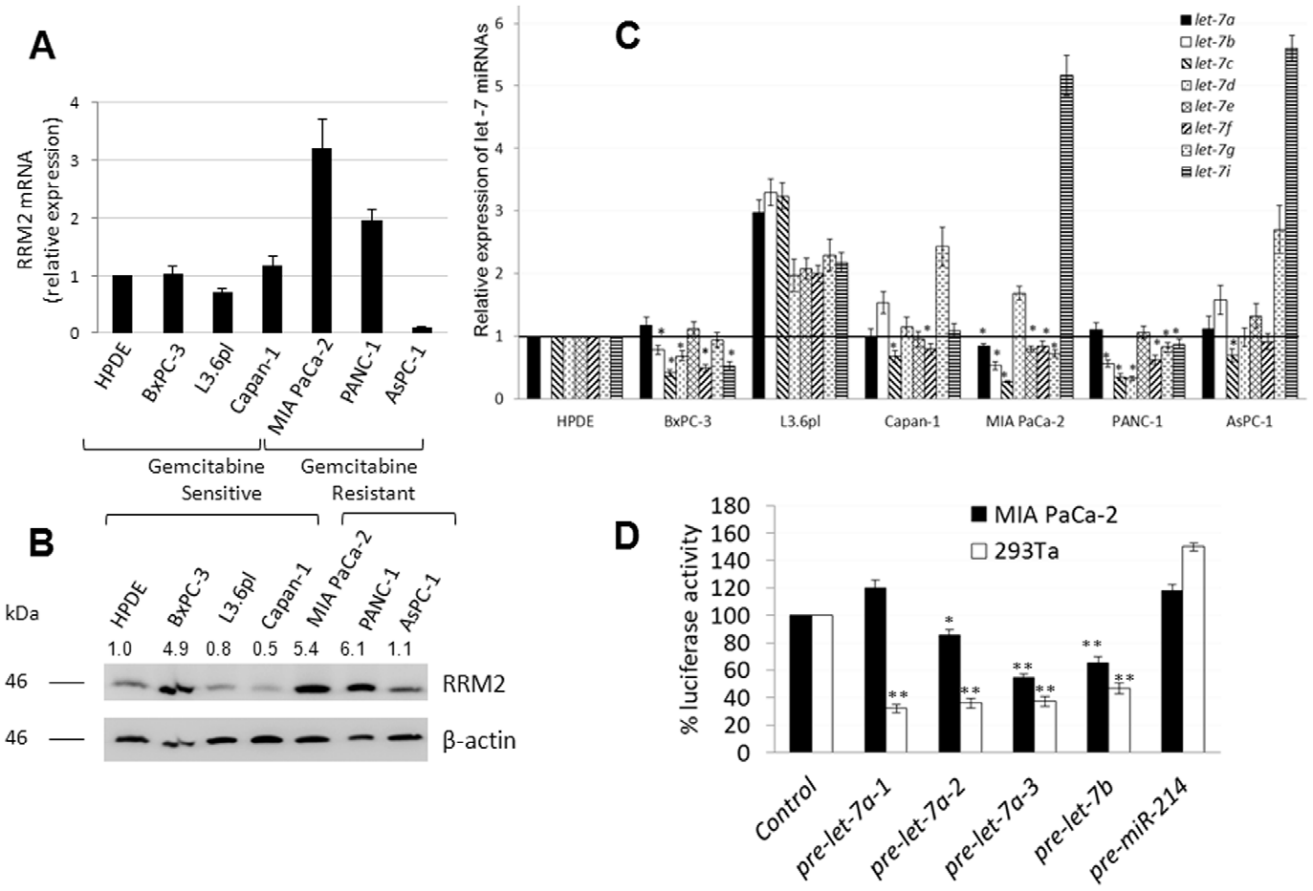
An inverse relation of RRM2 and *let-7 in* human pancreatic cancer cells. *A,* RRM2 mRNA expression in pancreatic cancer cell lines relative to expression identified in HPDE. *Columns,* mean of triplicate; *bars,* SD. n = 3. *B,* Western blotting analysis of RRM2 (∼45 kDa) and β-actin (45 kDa) in whole cell lysates of HPDE and pancreatic cancer cell lines. *C,* Expression of *let- 7* family members in pancreatic cancer cell lines relative to expression in HPDE. *Columns*, mean of triplicate; *bars*, SD. n = 3; *P<0.05. *D,* RRM2 is a direct target of *let-7*. 293TA and MIA PaCa-2 cells were virally infected for expression of precursors of *let-7a-1*, *let-7a-2*, *let-7a-3*, *let-7b*, and miR-214 (*negative control*) and subsequently transfected with a RRM2 3′ UTR luciferase reporter construct. Luciferase activities measured 36 h after transfection (normalized relative to renilla activity) were plotted. *Columns*, mean of triplicate; *bars*, SD. n = 3. *p<0.05, **p<0.01.

We first verified RRM2 expression in pancreatic cancer cell lines that were categorized earlier as inherently gemcitabine-sensitive or -resistant [23]. qRT-PCR (*Fig. 1A*) and Western blotting (*Fig. 1B*) analyses showed significantly higher RRM2 mRNA (2–3-fold) and protein (5–6-fold) expressions, respectively, in 2 out of the 3 gemcitabine-resistant cell lines studied (MIA PaCa-2 and PANC-1) as judged by comparisons with a normal human pancreatic ductal epithelial (HPDE) cell line. Conversely, comparable RRM2 expressions were identified between most gemcitabine-sensitive cell lines (L3.6pl and Capan-1) and HPDE (*Fig. 1A–B*). These results suggest that an overexpression of RRM2 is likely to play a role in gemcitabine chemoresistance in the majority of pancreatic cancer cell lines, if not all.

To assess the *let-7* control of RRM2 expression, we subsequently profiled the aforementioned cell lines for relative expression of all *let-7* family members by qRT-PCR. Interestingly, significantly lower expressions of most of the *let-7* miRNAs were observed only in cell lines with relatively greater RRM2 expression. MIA PaCa-2 exhibited reduced expression of *let-7a*, *let-7b*, *let-7c*, *let-7e*, *let-7f*, and *let-7g*, PANC-1 exhibited reduced expression of *let-7b*, *let-7c*, *let-7d*, *let-7g*, *let-7h*, and *let-7i*, and BxPC-3 exhibited reduced expression of *let-7b*, *let-7c*, *let-7d*, *let-7f*, and *let-7i* (*Fig. 1C*). None to only a few *let*-7 members had significantly reduced expressions in the remaining cell lines that expressed similar levels of RRM2 protein as HPDE (i.e., L3.6pl: none; AsPC-1: *let-7c*; Capan-1: *let-7c*, *let-7f*) (*Fig. 1C*). These results support an inverse relationship between RRM2 and *let-7* expression in pancreatic cancer cells.

We then tested the direct interaction of *let-7* with RRM2 by transfecting a luciferase-expression construct fused to the 3′ UTR of RRM2 into 293Ta (ATCC-CRL-9078) [23] and MIA PaCa-2 transiently overexpressing *let-7* members. While all *let-7* members significantly decreased luciferase expression in 239Ta cells, many *let-*7 members brought a significant decrease in luciferase expression in MIA PaCa-2 cells as well (*Fig. 1D*), suggesting that the direct binding of *let-7* to the RRM2 3′ UTR causes RRM2 repression. Finally, since *let-7* overexpression increases the G2/M fraction of fibroblasts [25] and RRM2 expression is specific to S-phase cells, we evaluated the role of *let-7* in reducing RRM2 expression by decreasing the proportion of MIA PaCa-2 in S-phase. Under these conditions, however, no prominent decreases in S-phase cells were observed in any of the pre-*let-7* overexpressing MIA PaCa-2 (*Fig. S1*). Taken together, these data likely suggest that *let-7* members may endogenously inhibit RRM2 expression by direct post-transcriptional repression in MIA PaCa-2.

### Human *let-7* Precursors Differentially Modify RRM2 Expression and Gemcitabine Chemosensitization in MIA PaCa-2

**Figure 2:**
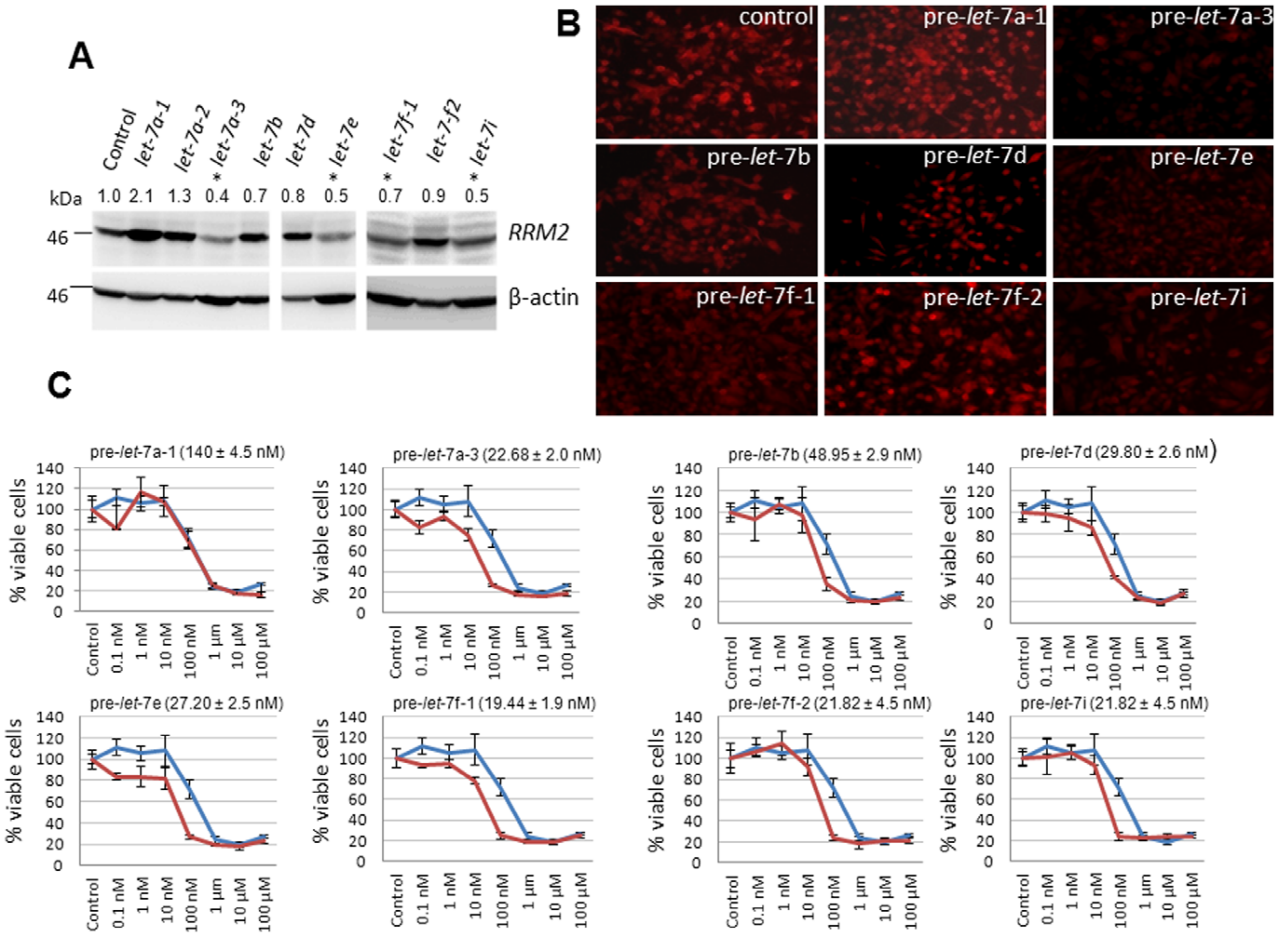
Differential RRM2 expression and gemcitabine chemosensitization by *let-7* precursors in MIA PaCa-2. *A*, Western blotting analysis of RRM2 (∼45 kDa) and β-actin (45 kDA) in whole cell lysates of MIA PaCa-2 overexpressing precursors of *let-7* family members. Ratios of RRM2 to β-actin band intensities (normalized to control) from three experiments are indicated (*top*). Asterisks indicate significant reductions (*p*<0.05) in RRM2 levels compared with control. *B*, Immunocytochemical detection of RRM2 in exponentially growing MIA PaCa-2 overexpressing pre-*let-7* family members. Original magnification, x20. *C*, MIA PaCa-2 cells stably overexpressing pre-*let-7* family members (*red*) or vector alone (*blue*) were treated with gemcitabine (0.1 nM to 100 µM), and percent inhibition of cellular proliferation was measured using an MTT assay. *Points*, mean of triplicate; *bars*, SE. n = 3. Gemcitabine IC_50_ estimations indicated (*parentheses*).

We next attempted to generate stable clones of MIA PaCa-2 that overexpresses one of ten human *let-7* precursors [27] to study their effects on RRM2 protein and chemosensitivity. We chose the MIA PaCa-2 cell line for these investigations because it exhibits low sensitivity to gemcitabine, especially at sub-confluent conditions, but expresses high levels of RRM2 (*Fig. 1B*) [23]. In addition, MIA PaCa-2 represents a poorly-differentiated pancreatic cancer cell model [23] and *let-7* plays critical roles in cellular differentiation. MIA PaCa-2 stably expressing pre-*let-7a-1*, pre-*let-7a-*2, pre-*let-7a-3*, pre-*let-7b*, pre-*let-7d*, pre-*let-7e*, pre-*let-7f-1,* pre-*let-7f-2*, and pre-*let-7i* were generated successfully by lentiviral gene transfer; however, repeated attempts to stably transduce pre-*let-7c* and pre-*let-7g* failed due to a lack of surviving colonies. Interestingly, Western blotting analysis showed significant reductions in RRM2 protein expression only in MIA PaCa-2 stably expressing pre*-let-7a-3*, pre*-let-7e*, pre*-let-7f-1*, and pre*-let-7i* but only minimally in MIA PaCa-2 cells expressing pre*-let-7b*, pre*-let-7d*, and pre*-let-7-f-2* cells (*Fig. 2A*). Surprisingly, the level of RRM2 protein increased in MIA PaCa-2 expressing pre-*let-7a-1* (*Fig. 2A*). Immunocytochemical analysis of these stable clones for RRM2 expression [26] confirmed these results (*Fig. 2B*). These data identify that RRM2 expressional outcomes significantly differ with the overexpression of specific pre-*let-7* subtypes in pancreatic cancer cells.

Since most *let-7* members [27] seemed to negatively influence RRM2 expression, we further investigated whether pre-*let-7* could augment chemosensitivity of MIA PaCa-2 to gemcitabine. Interestingly, significant reductions in gemcitabine cytotoxic IC_50_ estimations were identified in almost all pre-*let-7*-expressing MIA PaCa-2 stable clones generated with the only exception being pre-*let-7a-1* whose introduction brought no differences (*Fig. 2C*). In order to test whether the *let-7*-mediated increase in gemcitabine cytotoxicity was facilitated by RRM2 suppression, we overexpressed RRM2 cDNA with or without the 3′ UTR regions into MIA PaCa-2 expressing pre-*let-7a-3*. Our results identified lower gemcitabine cytotoxicity IC_50_ in cells expressing RRM2 with the 3′ UTR (69.34±3.4 nM) compared with those without the 3′ UTR (383.4±20.3 nM). These results suggest that the reduction in RRM2 protein as a result of pre-*let-7a-3* overexpression was facilitated by post-transcriptional repression of RRM2, although RRM2-independent mechanisms are likely to play predominant roles in other pre-*let-7*-overexpressing cells (e.g., pre-*let-7f-2*).

### Defective Processing of Pre-*let-7a-1* in MIA PaCa-2

**Figure 3:**
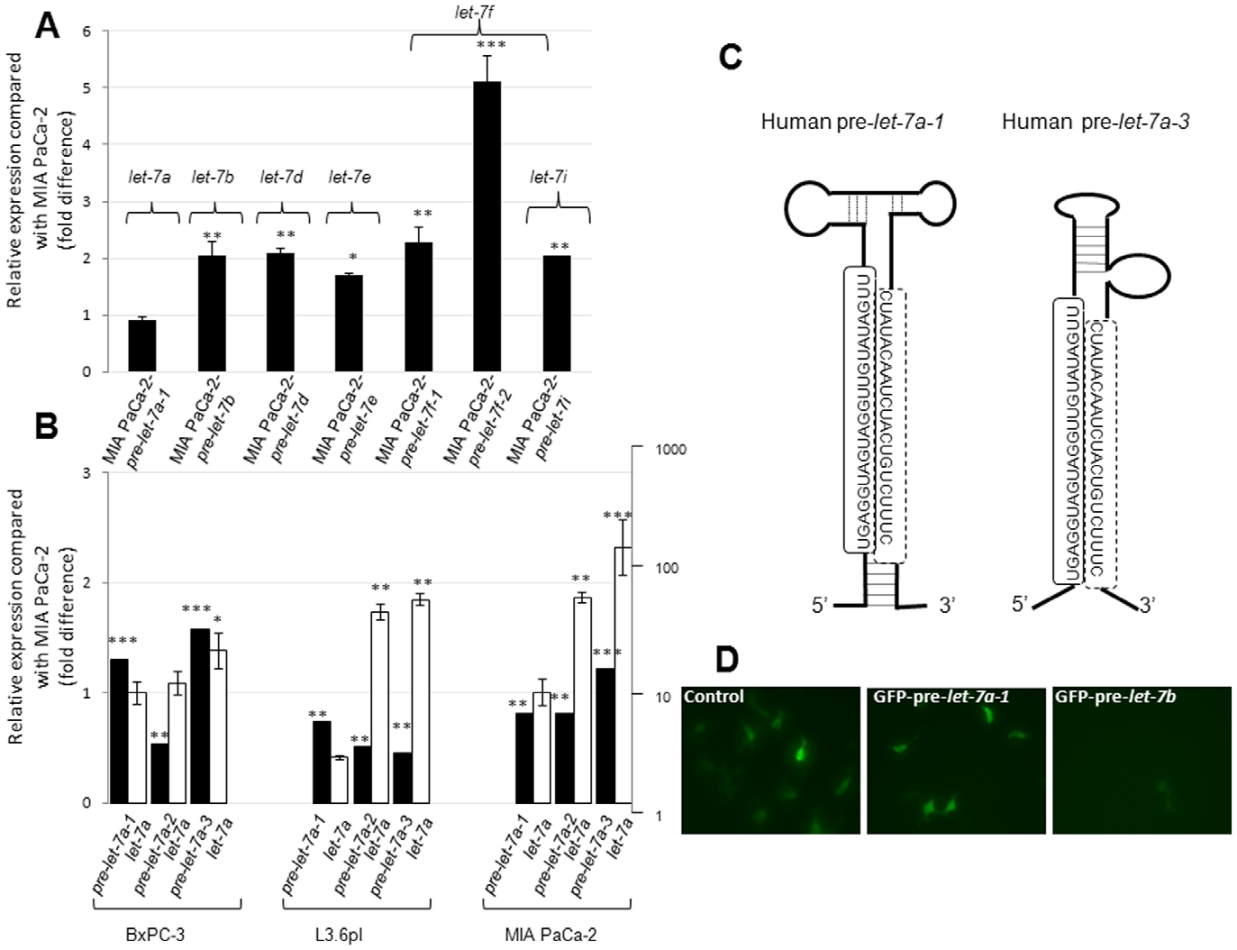
Defective processing of pre-*let-7a-1*, but not pre-*let-7a-3*, into *let-7a* in MIA PaCa-2. *A*, Relative expression of mature forms of *let-7* in MIA PaCa-2 stably expressing pre-*let-7* family members. *Columns,* mean of triplicate; *bars,* SD. n = 3. *B,* Relative expression of precursor (*filled bars*; *right axis*) and mature (*open bars*; *left axis*) *let-7* forms in pancreatic cancer cells transiently expressing *let-7a precursors*. *Columns,* mean of triplicate; *bars,* SD. n = 3. *C,* Schematic representation of the structures of pre-*let-7a-1* and pre-*let-7a-3*. Sequences of mature and passenger *let-7a* strands within the precursors are boxed in continuous or broken lines, respectively. *D,* Lack of complete cleavage of pre-*let-7a-1*-GFP mRNA in MIA PaCa-2 cells. MIA PaCa-2 cells were transiently transfected with either pmirGLO-G-Fud (control), pmirGLO-GFP-pre-*let-7a-1*, or pre-pmirGLO-GFP-pre-*let-7b* constructs, and GFP fluorescence was captured. Original magnification, x20. *p<0.05, **p<0.01, ***p<0.001.

Although we used pre-*let-7* members for generating all stable MIA PaCa-2 clones, functional RNA interference was expected to be mediated by the mature *let-7* miRNAs generated after a series of intracellular RNA processing events. Owing to differences in RRM2 expression and gemcitabine chemosensitization upon overexpression of pre*-let-7* members, we suspected that some of these precursors failed to process into mature *let-7* forms in MIA PaCa-2. Hence, we quantified relative mature *let-7* levels by qRT-PCR in various pre-*let-7-*expressing MIA PaCa-2 clones and compared them with mock-transduced MIA PaCa-2. Interestingly, significant increases (averages range from 2–5-fold) in mature *let-7* forms were identified in all pre-*let-7*-overexpressing cells tested, except for pre-*let-7-a-1*-overexpressing cells which did not show any alteration in mature *let-7a* levels (*Fig. 3A*).

Since defective miRNA processing machinery could downmodulate mature miRNAs [16], we next investigated whether such a defect is particularly affecting *let-7a* biogenesis in pancreatic cancer. As we did not observe an overexpression in mature *let-7a* in MIA PaCa-2 (*Fig. 3A*), we chose to investigate, in detail, the expression/processing of all three *let-7a* precursor forms (pre-*let-7a-1*, pre-*let-7a-2*, and pre-*let-7a-3*) that are derived from three separate genes (chromosomal locations 9q22.32, 11q24.1, and 22q13.31, respectively) [27]. Using qRT-PCR and primers that either flank the entire stem-loop structure (which detect miRNA precursors) or the mature sequence (which detects mature miRNA), we quantified the intracellular levels of all three *let-7a* precursors as well as mature *let-7a* in various pancreatic cancer cells transiently overexpressing pre-*let-7a-1*, pre-*let-7a-2*, or pre-*let-7a-3*.

While precursor forms were highly expressed in all three cases (3–38-fold increase), mature *let-7a* was only consistently increased in pre-*let-7a-3*-expressing cells but not in those expressing pre-*let-7a-1* (L3.6pl, MIA PaCa-2) (*Fig. 3B*). Cells expressing pre-*let-7a-2* showed intermediate levels (*Fig. 3B*). Even the increase in relative *let-7a* levels was only modest (∼2.2-fold in pre-*let-7a-3*-expressing MIA PaCa-2 cells). However, further analysis of the ratios of mature (*let-7a*) to precursor forms indicated a ∼16-fold reduction in the pre-*let-7a-1*-expressing MIA PaCa-2 cells when compared with pre-*let-7a-3*-expressing MIA PaCa-2 cells. These data identify profound defects in the complete processing of pre-*let-7a-1* but not pre-*let-7a-3* into their mature *let-7a* form in MIA PaCa-2, even though both are expected to generate the same mature form (i.e., *let-7a*; *Fig. 3C*). Finally, to directly corroborate the defective processing of pre-*let-7a-1* in pancreatic cancer cells, we transfected MIA PaCa-2 with constructs that express pre-*let-7a-1* fused to a GFP mRNA. If processing occurs, it is expected to induce cleavage of the GFP mRNA and reduce GFP fluorescence. As shown in *Fig. 3D*, a pre-*let-7a-1* fusion construct failed to undergo complete processing, but a control pre-*let-7b* fusion construct, which produced significantly higher mature *let-7* levels (*Fig. 3A*), did not. These results corroborate the defective processing of pre-*let-7a*-1 in pancreatic cancer cells.

### *let-7a* Processing Defects Progressively Increase with Pancreatic Cancer Cell Acquired Gemcitabine Resistance

**Figure 4:**
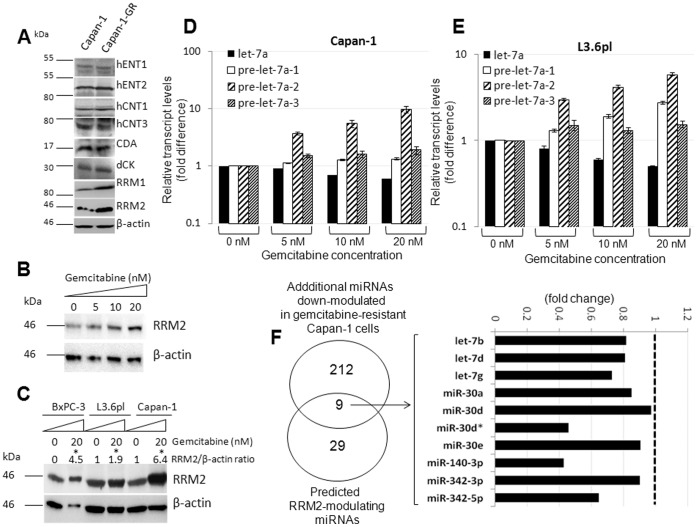
Acquired gemcitabine resistance is accompanied with RRM2 overexpression and defective *let-7a* precursor processing. *A,* Western blotting analysis of hENT1 (∼50–55 kDa), hENT2 (50 kDa), hCNT1 (72 kDa), hCNT3 (77 kDa), CDA (55 kDa), dCK (30 kDa), RRM1 (94 kDa), RRM2 (45 kDa), and β-actin (45 kDA) levels in whole cell lysates of Capan-1 and Capan-1-GR cells. *B*, RRM2 protein increased in a gemcitabine dose-dependent fashion in Capan-1-GR cells. *C,* Western blotting analysis of RRM2 (∼45 kDa) and β-actin (45 kDA) levels in cells with acquired gemcitabine resistance. Ratios of RRM2 to β-actin band intensities (normalized to untreated cells) from three experiments are indicated (*top*). Asterisk indicates significantly higher RRM2 expression in gemcitabine-resistant cells (*p*<0.05) compared with untreated cells. *D* and *E,* Relative expression of precursor and mature *let-7a* in Capan-1 (*D*) and L3.6pl (*E*) cells induced to acquire gemcitabine resistance. *Columns,* mean of triplicate; *bars,* SD. n = 3. *F*, Differential miRNA expression in Capan-1-GR compared with Capan-1. Putative RRM2-modulating miRNAs and their extent of reduction in Capan-1-GR cells are shown (*right*).

Next, to investigate the role of the RRM2-*let-7* interplay on acquired gemcitabine chemoresistance, we first chronically treated gemcitabine-sensitive human pancreatic cancer cell lines (L3.6pl and Capan-1) [23] with escalating doses of gemcitabine (5–20 nM), and surviving clones with characteristics of acquired gemcitabine resistance were investigated. In Capan-1 gemcitabine-resistant cells (Capan-1-GR), we assessed expressional alterations of candidates known to directly impact gemcitabine chemosensitivity (*Fig. 4A*). Specifically, we tested proteins involved with gemcitabine transport (i.e., hENT1, hENT2, hCNT1, and hCNT3), phosphorylation (i.e., deoxycytidine kinase; dCK), metabolism (i.e., cytidine deaminase; CDA), and cytotoxicity (i.e., ribonucleotide reductase subunit 1 (RRM1) and RRM2) by Western blotting analyses [34]. Of all, RRM2 showed maximal changes in Capan-1-GR (20 nM) with RRM2 protein increased by ∼6.4-fold (*Fig. 4A and 4C*). Furthermore, the increases in RRM2 protein were directly correlated with the increasing doses of gemcitabine used to develop resistance (*Fig. 4B*). Similar results were obtained with L3.6pl-GR and BxPC-3-GR clones (*Fig. 4C*). The levels of other candidates showed either moderate or no change (*Fig. 4A*). A significant increase (∼3.8-fold) in the RRM1 protein was also observed but to a lesser degree than RRM2.

Since not all RRM2 protein induction (>5-fold in Capan-1) could be fully accounted for by the increase in RRM2 transcripts (≤2-fold), we subsequently tested whether a decrease in *let-7*-mediated post-transcriptional repression of RRM2 was promoting acquired resistance. To test this, we quantified and compared levels of the three *let-7a* precursors and mature *let-7a* between Capan-1-GR and L3.6pl-GR cells and their untreated WT counterparts. Although moderate, the mature *let-7a* progressively decreased, and precursor *let-7a* forms severely accumulated in a progressive, dose-dependent fashion in both Capan-1-GR (*Fig. 4D*) and L3.6pl-GR (*Fig. 4E*). Maximal accumulation of precursors was noticed with pre-*let-7a-2* (Capan-1-GR) and pre-*let-7a-1* (Capan-1-GR and L3.6pl-GR). These results clearly support the occurrence of defective *let-7a* precursor processing with acquired nucleoside analog chemoresistance in pancreatic cancer cells.

Since TargetScanHuman 5.1 predicted multiple miRNAs to bind to RRM2, we also tested the possible involvement of additional miRNAs in increasing RRM2 expression in Capan-1-GR cells. Profiling of Capan-1 and Capan-1-GR cells using a miRNA array for differential expression identified 212 miRNAs (out of 494 miRNAs) showing a >2-fold reduction in Capan-1-GR cells (*Fig. 4F*). Comparisons with computationally predicted RRM2 targeting miRNAs identified that in addition to reduction in several *let-7* members, mir-140-3p, the miR-30 family, and miR-342-5p were also found to potentially contribute to the overall induction of RRM2 expression in Capan-1-GR cells (*Fig. 4F*).

### Several Novel RNA-binding Proteins Influence Mature *let-7a* Biogenesis in MIA PaCa-2

**Figure 5:**
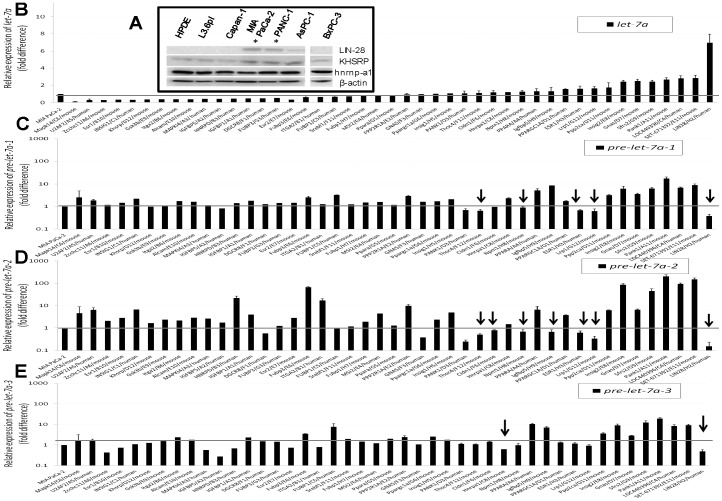
Screening for putative *let-7a* biogenesis regulators in MIA PaCa-2. *A,* Western blotting analysis of LIN-28A (26 kDa), hnRNP-A1 (36 kDa), KHSRP (∼66–70 kDa), and β-actin (45 kDA) in whole cell lysates of normal and cancerous pancreatic cells. Asterisk indicates significantly higher LIN-28 expression (*p*<0.05) compared with that identified in HPDE from three experiments. *B–E*, Screening for regulators of *let-7a* biogenesis. MIA PaCa-2 cells were infected with lentiviruses harboring shRNAs for putative RNA processing proteins, and the relative levels of mature *let-7a* (*B*) and three precursors of *let-7a* (*C–E*) in stable clones were plotted. Data are mean±SD. n = 3. Arrows indicate the candidates showing reductions in precursor *let-7a* with a concomitant increase in mature *let-7a*.

In order to study whether misexpression of regulatory proteins were responsible for the observed defects in *let-7a* processing in pancreatic cancer cells, we first investigated the expression of LIN-28, a pluripotent stem cell protein that has been well-established to negatively regulate *let-7* biogenesis [28], [29]. Western blotting analysis identified detectable levels of LIN-28 protein in MIA PaCa-2, PANC-1, and AsPC-1 (gemcitabine-resistant) but only minimally or at almost undetectable levels in HPDE, L3.6pl, and Capan-1 (gemcitabine-sensitive) (*Fig. 5A*). Levels of two other known regulators of *let-7* biogenesis, KHSRP (positive regulator) and hnRNP-A1 (negative regulator) [30], were not notably different between the various pancreatic cancer cell lines tested and HPDE (*Fig. 5A*). These data persuaded us to test for the existence of additional regulators of *let-7* biogenesis in drug-resistant pancreatic cancer cells.

To extend this search, we used a lentiviral-based shRNA library screening approach to generate stable MIA PaCa-2 clones that produced RNA interference for many putative RNA binding/processing proteins. We successfully generated stable MIA PaCa-2 clones for 45 out of 96 putative RNA-binding proteins tested using a GFP coexpression strategy that allowed us to confirm expression of shRNAs for a given target. Subsequently, variations in both precursor and mature *let-7a* forms were tested in these 45 clones. Twenty-two shRNAs showed >2-fold difference in mature *let-7a* levels when compared with mock-transduced clones (*Fig. 5B*). Specifically, shRNAs against Insig-2, Gnas, Sfrs2, PANK-1, LOC-440396, SET, and LIN-28 showed a >2-fold increase in mature *let-7a* levels (*Fig. 5B*). When silencing Insig-2, LOC-440396, Gnas, PANK-1, and SET, increases in mature *let-7a* levels were associated with concurrent increases in three *let-7a* precursors; however, the LIN-28 silencing-mediated increase in mature *let-7a* levels was associated with a decrease in all three precursors (*Fig. 5C–E*). In addition, Thoc4, Cldn1, Npm1, Igfbp5, ESR1, and Lrp1 also showed moderate but significant increases in *let-7a* levels with concomitant reductions in one or more pre-*let-7a* levels (*Fig. 5C–E*). These data identified multiple RNA binding proteins influencing mature *let-7a* levels in pancreatic cancer cells.

### LIN-28 and SET Oncoprotein Affect Mature *let-7* Expression and Chemosensitization Differentially in Gemcitabine-sensitive Versus Gemcitabine-resistant Cells: Pronounced Growth Suppression with SET knockdown

**Figure 6:**
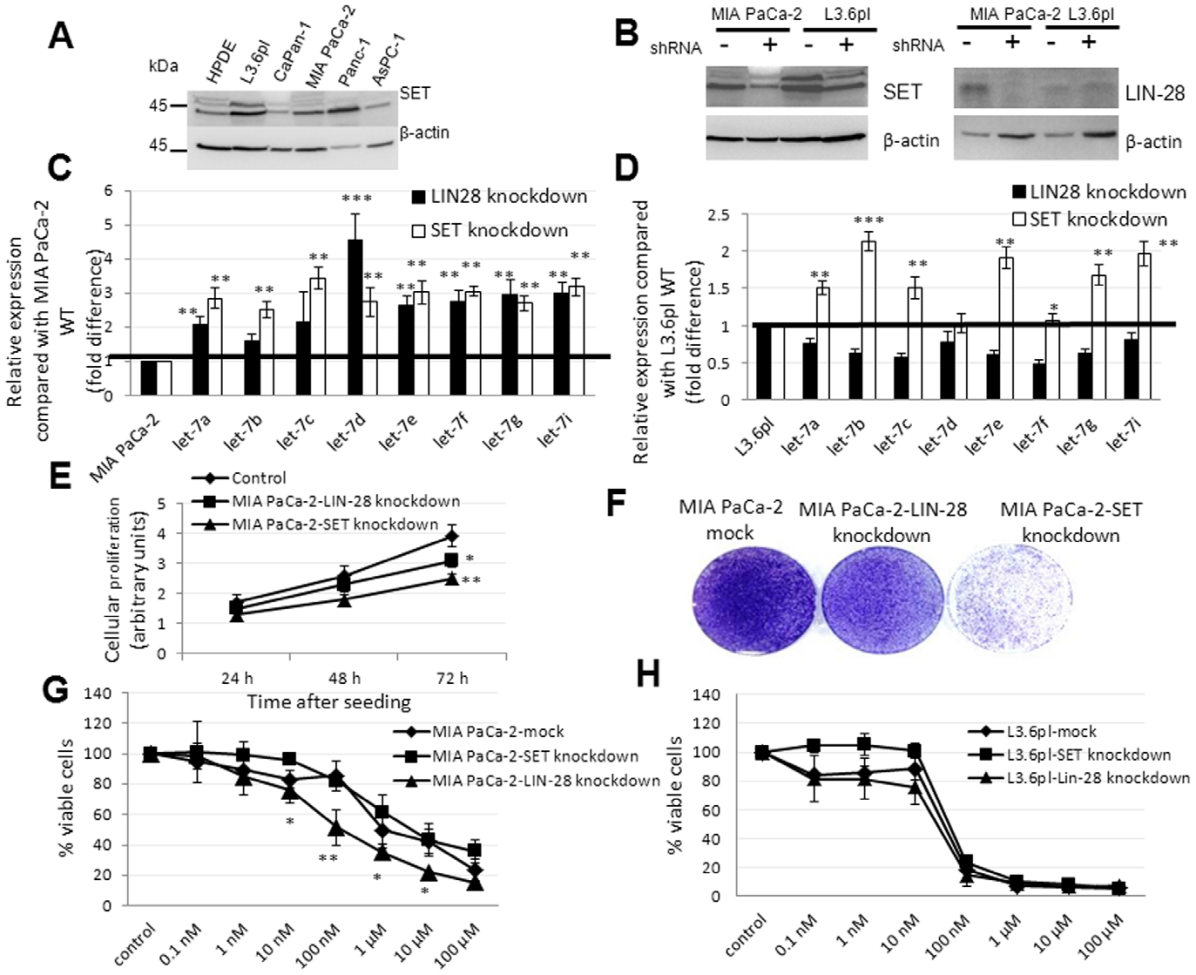
Silencing of LIN-28 and SET showed differential *let-7* biogenesis, growth, and gemcitabine chemosensitivity effects. *A,* Overexpression of SET in pancreatic cancer. Western blotting analysis of SET (39 kDa) and β-actin (45 kDa) in HPDE and pancreatic cancer cell lines. *B,* Western blotting analysis of SET (39 kDa) or LIN-28 (26 kDa) in MIA PaCa-2 expressing shRNAs for the respective proteins. Mock-transduced cells were used for comparison, and β-actin (45 kDa) was used as a loading control. *C and D*, Relative expression of mature *let-7* members in LIN-28- (*filled bars*) or SET-silenced (*open bars*) MIA PaCa- 2 (*C*) and L3.6pl (*D*). *Columns,* mean of triplicate; *bars,* SD. n = 3. *E* and *F*, Relative cellular proliferation (*G*) and colony-formation (*H*) capacities of control and LIN-28- or SET-silenced MIA PaCa-2 compared with mock-transduced MIA PaCa-2. *G* and *H,* 3×10^3^ control and LIN-28- or SET-silenced MIA PaCa-2 (*E*) and L3.6pl (*F*) were treated with gemcitabine (0.1 nM to 100 µM), and percent inhibition of cellular proliferation measured by an MTT was plotted. *Points*, mean of triplicate; *bars*, SD. n = 3. *p<0.05, **p<0.01, ***p<0.001.

We next examined whether manipulating LIN-28 and SET, which produced the highest changes in *let-7a* levels in our screen (*Fig. 5B*), could influence the biogenesis of various *let-7* miRNAs. We decided to examine all of the 10 human *let-7* members for their potential roles as chemosensitization factors [17], [31]–[33]. The analysis was performed in both gemcitabine-sensitive (L3.6pl) and gemcitabine-resistant (MIA PaCa-2) pancreatic cancer cells. SET itself, as judged by Western blotting, was found to be overexpressed in most of the pancreatic cancer cell lines when compared with HPDE (*Fig. 6A*). While qRT-PCR showed that knockdown of LIN-28 (*Fig. 6B*) only increased mature *let-7* levels in MIA PaCa-2 (8 out of 8 *let-7* members) and not L3.6pl, knockdown of SET (*Fig. 6B*) increased the levels of *let-7* members in both L3.6pl (6 out of 8 *let-7* members) and MIA PaCa-2 (8 out of 8 *let-7* members) (*Fig. 6C–D*). This was consistent with the lack of detectable LIN-28 expression in L3.6pl and presence of SET expression in both L3.6pl and MIA PaCa-2 (*Fig. 5A* and *6A*). Interestingly, we observed both SET and LIN-28 knockdown to significantly reduce MIA PaCa-2 proliferation and colony-forming abilities with the SET knockdown to produce a comparatively higher growth suppressive effect than the LIN-28 knockdown (*Fig. 6E–F*). Further, MTT analyses indicated that SET knockdown had no significant effect on gemcitabine chemosensitivity in either L3.6pl or MIA PaCa-2 (*Fig. 6G–H*), whereas LIN-28 knockdown selectively increased gemcitabine chemosensitivity in MIA PaCa-2 (IC_50_∶102.34±5.2 nM) and not L3.6pl (*Fig. 6G–H*). Together, these results identify LIN-28 and SET oncoprotein to differentially modulate *let-7* expression and chemosensitivity in gemcitabine-sensitive versus –resistant pancreatic cancer cells with LIN-28 selectively influencing gemcitabine chemosensitivity in poorly differentiated pancreatic cancer cells (i.e., MIA PaCa-2).

### Defective Processing of *let-7a* Precursors and RRM2 Overexpression Identified in Patient-derived PDAC Tissues

**Figure 7:**
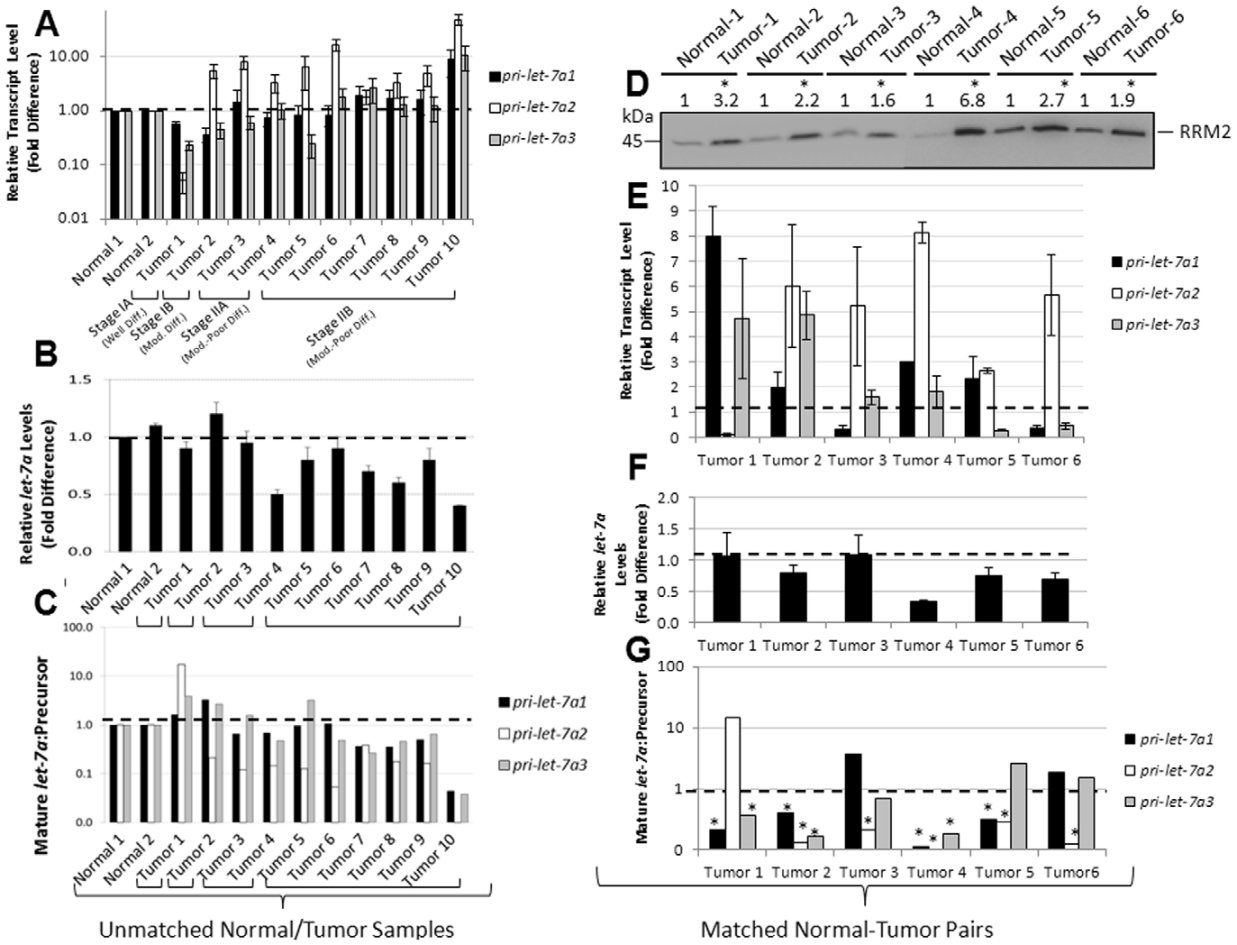
Defective processing of *let-7a* precursors and RRM2 overexpression in resected human PDAC tissues. *A* and *B,* Relative expression of primary *let-7a* transcripts (*A*) and mature *let-7a* (*B*) in 2 normal pancreatic tissues and 10 PDAC samples representing various tumor stages. *C*, Ratios of mature to precursor *let-7a* transcripts calculated from data points in *A* and *B*. D, Western blotting analysis of RRM2 (45 kDa) in total lysates (50 µg) of 6 matched normal-PDAC pairs. Ratios of RRM2 band intensities in PDACs compared to matched normal tissues indicated (*top*). Asterisk indicates significantly higher RRM2 expression in PDAC tissues (*p*<0.05) compared with matched normal tissues. *E* and *F,* Relative expression of primary *let-7a* transcripts (*E*) and mature *let-7a* (*F*) in matched normal-PDAC pairs. *G*, Ratios of mature to precursor *let-7a* transcripts calculated from data points in *E* and *F*. Asterisk indicates significantly lower mature to precursor *let-7a* ratio in PDAC tissues (*p*<0.05) compared with matched normal tissues.

*In vitro* results so far demonstrated defective processing of *let-7a* precursors in poorly differentiated pancreatic cancer cells with critical influences on growth and chemosensitivity. On the basis of this conclusion, we hypothesized that the *let-7a* processing defects would be present in poorly differentiated, high-grade pancreatic tumors. To validate this clinical relevance, we quantified the three precursor *let-7a* forms and mature *let-7a* in resected pancreatic ductal adenocarcinoma (PDAC) tissues representing four different stages (Stages IA, IB, IIA, and IIB) and varying degrees of differentiation (well-differentiated, moderately differentiated, moderately-poorly differentiated) (*See Table S1)*. qRT-PCR data from PDAC tissues were compared with the data obtained from two unmatched (i.e., derived from different donors), normal pancreatic tissue samples (*Fig. 7A*–*C*). These data indicated the mature *let-7a* levels to inversely correlate with the stages of PDAC with maximal reductions in *let-7a* noted in stage IIB, moderately-poorly differentiated PDAC tissues (the most severe stage examined) (*Fig. 7B*). Conversely, pri-*let-7a* levels were found to be significantly increased in Stage IB, Stage IIA, and Stage IIB (moderately to poorly differentiated) PDAC tissues (*Fig. 7A*). Consequently, the ratios of mature *let-7a* to pre*-let-7a* forms were found to be highly reduced in Stage IB, Stage IIA, and Stage IIB PDAC tissues (*Fig. 7C*) corroborating the increasing defects in pre-*let-7a* processing with pancreatic tumor progression.

Next, to investigate the correlation between defective *let-7* processing and RRM2 expression, we profiled 6 matched normal and PDAC tissues (i.e., derived from the same donors) for *let-7* and RRM2 expressions. Significant overexpression of RRM2 protein was identified in all 6 out of 6 matched PDAC samples (*Fig. 7D*), and defective processing of one or more *let-7a* precursors was also clearly identified in all 6 PDAC tissues (*Fig. 7E*–*G*). Furthermore, in both matched and unmatched PDAC tissues, reduction in precursor processing (>2-fold accumulation of the precursor form) to mature *let-7a* was more frequently noticed with pre-*let-7a-2* (4 out of 6 matched PDAC tissues; 9 out of 10 unmatched PDAC tissues) and pre-*let-7a-1* (4 out of 6 matched PDACs; 5 out of 10 unmatched PDACs) than that observed with pre-*let-7a-3* (2 out of 6 matched PDACs; 3 out of 10 unmatched PDACs). Taken together, these data identify defective processing in a rank order of pre-*let-7a-2*>pre-*let-7a-1*>pre-*let-7a-3* and the defects to follow a trend with increased RRM2 expression in human PDAC tissues.

## Discussion

Our study identified reduced *let-7* expression to contribute to the RRM2-mediated inherent chemoresistance in poorly differentiated pancreatic cancer cells. In addition, distinct *let-7* precursors were identified to improve chemosensitization in gemcitabine-resistant pancreatic cancer cells partially via post-transcriptional repression of RRM2. Varied consequences upon overexpressing mammalian *let-7* precursors in MIA PaCa-2, in particular the effects on RRM2 expression and gemcitabine chemosensitivity, suggest the existence of intricately controlled mechanisms. A striking example within *let-7a* precursors is that pre-*let-7a-3* reduced RRM2 expression and improved gemcitabine chemosensitivity, whereas pre-*let-7a-1* and pre-*let-7a-2* induced RRM2 expression with no significant reductions in gemcitabine chemosensitivity. Variations in the post-transcriptional processing of pre-miRNAs into mature forms, in part, provide explanations for the antagonistic actions of these precursors. Our study also elucidates several RNA processing proteins, including SET oncoprotein and LIN-28, to disparately modulate mature *let-7* biogenesis and chemosensitivity in gemcitabine-sensitive- versus –resistant pancreatic cancer cells. Overall, these data expand our current understanding of *let-7* regulation of growth control in pancreatic cancers.

Activation of RR was shown to be one of the key determinants of tumor growth, invasion, and chemoresistance to nucleoside analogs in solid tumors [10], [35]. While gene amplification, transcriptional activation, and allosteric activation of RRM2 [3]–[5] are some of the known mechanisms for RR induction in chemoresistant cancers, the possibility for miRNA regulation of RRM2 expression has never been investigated. Identification of the interaction between *let-7* miRNA and the 3′ UTR of RRM2 transcripts and the concomitant decrease in RRM2 protein expression in the absence of prominent cell cycle alterations provide supportive evidence for the *let-7*-mediated post-transcriptional repression of RRM2. Nevertheless, pre-*let-7*-mediated alterations in RRM2 expression may not be just because of a simple translation inhibition process. For instance, unlike the majority of *let-7* precursors that decreased RRM2 expression, pre-*let-7a-1* and pre*-let-7a-2* stimulated RRM2 expression while other *let-7* members (i.e., pre-*let-7f-2*) did not significantly alter RRM2 levels. Although our data unequivocally identify defective processing of pre*-let-7a-1* and pre*-let-7a-1* as a key reason for the observed failure to increase mature *let-7a* expression, it was unexpected to find RRM2 expression increased in pre*-let-7a-1*-overexpressing cells. Likewise, even when mature *let-7* increased with overexpression of other precursors (i.e., pre-*let-7d*, pre-*let-7f-2*), no alterations in RRM2 expression were observed. While the precise reasons for such discrepancies are unclear, several hypotheses can be put forth in explaining these outcomes. First, the diverse targets, even for closely-related miRNAs such as those within the *let-7* family [27], can evoke markedly different cellular outcomes based on the collective effect of their individual targets. Second, certain miRNAs, including members of the *let-7* family, have been shown to activate rather than suppress target gene expressions under specific cellular environments [36]. Third, since precursor *let-7* forms are also capable of binding to target transcripts similar to mature *let-7* [37], increased levels of pre-*let-7a-1*, even in the absence of mature *let-7*, could force incorporation of RRM2 into RISC, perhaps modulating gene expression. Evidently, we noticed pre-*let-7a-1* to moderately activate RRM2 expression in reporter-based RNA interference assays in MIA PaCa-2 (*Fig. 1* *D*) despite its inability to process pre-*let-7-a-1* to mature *let-7a*. Fourth, *let-7* could also act on transcriptional factors, proteasomal machinery, cell cycle check points, DNA replication/repair enzymes, etc. which can indirectly influence RRM2 expression [9], [26], [38]–[40]. Finally, RRM2 may not be a global determinant of drug-resistance in pancreatic cancer cells, in which case the proposed *let-*7-RRM2-chemoresistance axis may not be as effective as expected in RRM2-dependent resistance. For instance, gemcitabine-resistant AsPC-1 expressed low levels and gemcitabine-sensitive BxPC-3 expressed high levels of RRM2 protein. Similarly, gemcitabine-resistant MIA PaCa-2 and PANC-1 expressed very high levels of endogenous *let-7i* yet exhibited high levels of resistance to gemcitabine. Hence, RRM2-independent drug-resistance mechanisms cannot be negated while considering nucleoside analog chemosensitization in pancreatic cancer cells. In this regard, induction of the non-regulatory RRM1 subunit, expression of anti-apoptotic proteins, activation of cell survival genes, and induction of drug-efflux proteins (e.g., MRPs) have been shown to modulate gemcitabine chemosensitization in drug-resistant pancreatic cancer cells [41]–[45].

Investigating expressional alterations of *let-7* miRNAs in pancreatic cancer cells led to the identification of the influence of various RNA binding proteins in these processes. A direct role of LIN-28, a zinc finger protein that promotes pluripotency in embryonic stem cells [28], [29], was readily evident in the defective processing of *let-7a* as observed by increases in mature *let-7* levels upon LIN-28 knockdown and the concurrent enhancement of chemosensitivity. The expression of LIN-28 exclusively in poorly differentiated pancreatic cancer cells even suggested a role for the stem-cell characteristics of these cells in determining chemoresistance and potential avenues for utilizing this circuit to improve drug sensitivity. Further, our shRNA-based gene-silencing screen for novel regulators of pre-*let-7a-1* biogenesis brought several novel aspects to light. It displayed several putative candidates that could have a direct impact on post-transcriptional *let-7* processing (Thoc4, Cldn1, Npm1, Igfbp5, ESR1, Lrp1 and LIN-28; *Fig. 4C–E*). While analysis of the ratios of precursor to mature *let-7a* indicated that all three *let-7a* precursors were subject to a certain level of post-transcriptional processing, pre-*let-7a-2* and pre-*let-7a-3* were found to be the most and east regulated by this step, respectively. These data along with the recent identification of epigenetic silencing of pre-*let-7a-3* in other solid tumors [46], [47] suggest that regulation of pre-*let-7a-3* occurs before the post-transcriptional stage―perhaps at the transcriptional stage. Collectively, these data suggest pre-*let-7a-3* may be likely to act as one of the preferable candidates among *let-7a* precursors for therapeutic selections against pancreatic cancer.

Our studies also focused on the role of a novel candidate, SET oncoprotein, an inhibitor of protein phosphatase 2A (PP2A) whose role in pancreatic cancer was untested [48]. Our data showed high expression levels of SET oncoprotein in many of the pancreatic cancer cell lines; to our knowledge this is the first report demonstrating SET overexpression in pancreatic cancer. Silencing SET not only increased mature *let-7a* levels but also other members within the *let-7* family in both poorly differentiated MIA PaCa-2 and well-differentiated L3.6pl. It is likely that SET inhibits the transcription of many miRNAs, perhaps including tumor suppressors such as *let-7*, while silencing of SET removes this block. The observed increase in both precursor and mature *let-7* levels upon SET knockdown supports this hypothesis. Our data also identify SET knockdown to significantly reduce proliferation and colony-forming abilities of MIA PaCa-2 but did not improve chemosensitivity. We speculate that the profound growth arrest mediated by SET knockdown hinders gemcitabine activation since nucleoside analogs are known to semi-selectively target rapidly proliferating cells to induce cytotoxicity [23]. Nevertheless, the tumor suppressive role of SET in pancreatic cancer itself is worth considering for further evaluations.

In summary, RRM2 was found to be a key determinant of both inherent and acquired gemcitabine with reduced *let-7* expression likely to contribute to RRM2-mediated inherent chemoresistance in poorly differentiated pancreatic cancer cells. Besides several possibilities, alterations in *let-7* processing machinery were found to influence the levels of mature *let-7* as well as nucleoside analog chemoresistance in tumor cells. MicroRNAs are clearly emerging as a next generation therapeutic [49] and are in early clinical trials for the treatment of human diseases [50]. Our findings that *let-7* is capable of influencing gemcitabine chemosensitivity along with its tumor suppressive and differentiation-promoting functions in solid tumors extend its promise as a therapeutic candidate for pancreatic cancer. However, the ability of pancreatic cancer cells to restore or augment mature *let-7* expression must be carefully considered when choosing *let-7* as a therapeutic candidate. For example, the direct introduction of mature *let-7* forms is likely to bring enhanced outcomes in a heterogenic tumor population than the pre-*let-7* forms. Likewise, careful selection of pre-*let-7* subfamilies can also overcome defects associated with *let-7* processing machinery in pancreatic cancer cells. Furthermore, *let-7* regulatory proteins can also be targeted. Future studies, especially in animal models, are expected to improve the collective understanding of *let-7* cancer biology and its therapeutic applications in solid tumors.

## Supporting Information

Figure S1 **Lack of cell cycle changes in MIA PaCa-2 expressing pre-** ***let-7*** **members.** MIA PaCa-2 cells transiently infected with lentiviruses harboring empty (*control*) or various pre*-let-7* members were subjected to cell cycle analysis (48 h after transfection) as described earlier [23]. The percentages of cells in the various stages of the cell cycle are indicated.
(TIF)

Table S1 **Demographic and clinical information for the resected, unmatched human PDAC tissues.** The table includes information on age, sex, country, height, weight, BMI, confirmatory diagnosis, tumor stage, metastatic presence, smoking use, alcohol use, clinical and serological testing results, and medication use.
(XLSX)
