# Supplementary figures and images for: Differential Processing of let-7a Precursors Influences RRM2 Expression and Chemosensitivity in Pancreatic Cancer: Role of LIN-28 and SET Oncoprotein
Source: PLoS One. 2013 Jan 15;8(1):e53436. doi: 10.1371/journal.pone.0053436 (PMC3546076; doi:10.1371/journal.pone.0053436)

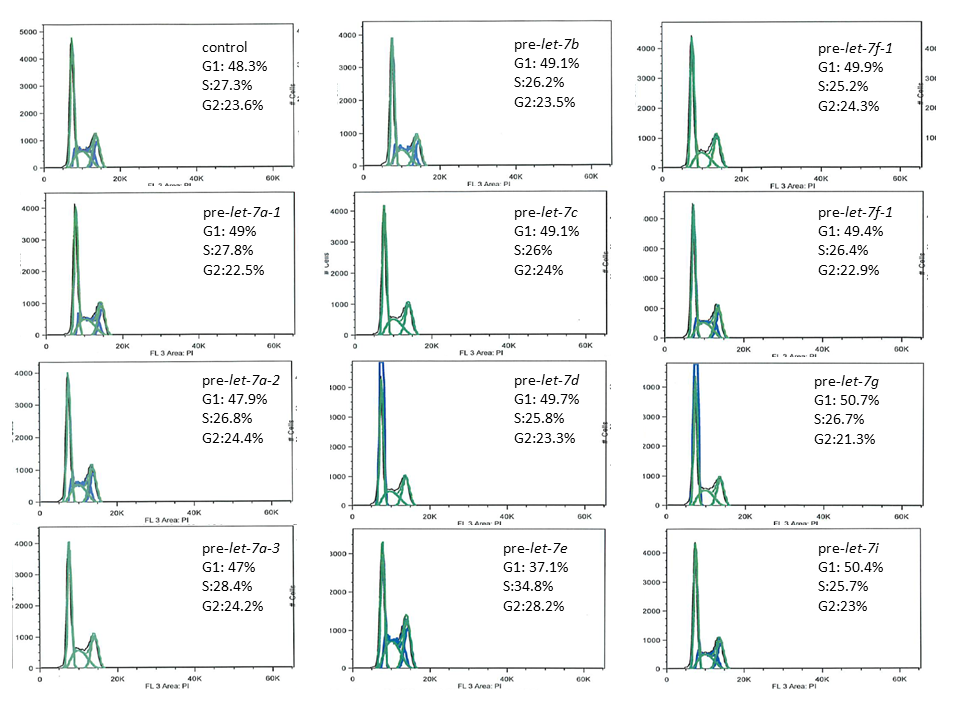

Supplement: Figure S1 — Lack of cell cycle changes in MIA PaCa-2 expressing pre- let-7 members. MIA PaCa-2 cells transiently infected with lentiviruses harboring empty (control) or various pre-let-7 members were subjected to cell cycle analysis (48 h after transfection) as described earlier [23]. The percentages of cells in the various stages of the cell cycle are indicated. (TIF) [file pone.0053436.s001.tif]
